# Contraceptive use, prevalence and predictors of pregnancy planning among female sex workers in Uganda: a cross sectional study

**DOI:** 10.1186/s12884-019-2260-4

**Published:** 2019-04-08

**Authors:** Justine Nnakate Bukenya, Rhoda K. Wanyenze, Geraldine Barrett, Jennifer Hall, Fredrick Makumbi, David Guwatudde

**Affiliations:** 1 School of Public Health, College of Health Sciences Makerere University, P.O. Box 7072, Kampala, Uganda; 2 Institute for Women’s Health, University College London, London, UK

**Keywords:** Female sex workers, Pregnancy planning, Predictors, Uganda, Low income countries

## Abstract

**Background:**

Unintended pregnancies are associated with negative consequences to both mother and baby. Female Sex Workers (FSWs) are at high risk of unintended/unplanned pregnancies. However, prevalence of pregnancy planning and its predictors among FSWs has not been comprehensively investigated. This study was designed to determine contraceptive use, the prevalence, and predictors of pregnancy planning among FSWs in Uganda.

**Methods:**

In this cross-sectional study, 819 FSWs attending most at risk populations initiative (MARPI) clinics were recruited using systematic sampling and interviewed with a pretested questionnaire that included collection of data on pregnancy intention using the London Measure of Unplanned Pregnancy (LMUP). Data were analysed using STATA version 14.0. Multinomial logistic regression model was used to identify predictors of pregnancy planning,

**Results:**

Of the 819 study participants, only 90 (11.0%) had planned pregnancies. Overall, 462 (56.4%) were hazardous alcohol users and 335 (40.9%) abused drugs; 172 (21.0%) had been raped in the last 2 years and 70 (40.7%) of these accessed emergency contraception post-rape. Dual contraception use (condom and other modern method) was 58.0%.

Having a non-emotional partner as a man who impregnated the FSW compared to emotional partner was significantly associated with less planned relative to unplanned pregnancy, (aRR = 0.15 95%Cl =0.08, 0.30), so was lack of reported social support compared to support from friends, (aRR = 0.44; 95% CI = 0.22–0.87), keeping all factors constant in the model. Being raped (aRR = 0.51; 95% CI = 0.31–0.84) or abuse of substances (aRR = 0.65; 95% CI = 0.45–0.93) were significantly associated with lower ambivalence relative to unplanned pregnancy but not with planned relative to unplanned pregnancy.

**Conclusion:**

Compared to women in the general population, pregnancy planning was low among FSWs amidst modest use of dual contraceptive. There is an urgent need to promote dual contraception among FSWs to prevent unplanned pregnancies especially with non-emotional partners, drug users, and post-rape.

## Background

Pregnancy planning is an important public health practice that should be promoted among women of reproductive age. Pregnancy planning involves, to a greater or lesser extent, the conscious choice to become pregnant and is a necessary component in the adoption of healthier lifestyles before conception that are associated with positive maternal and child outcomes [1]. On the contrary, unplanned pregnancies are associated with poor economic, social, and health consequences. Such negative outcomes include low-birth weight and increased risk of infant mortality [2], and among women abortion-related mortalities [3] and financial expenses while procuring abortion and care [4, 5]. Planning pregnancies provides an opportunity to harness the biomedical, behavioural and social health interventions to improve the health status of women in preparation for safe conception [6]. This contributes to better maternal and child health outcomes.

Though there is a challenge in estimating the actual number of Female Sex Workers (FSWs) globally, available data indicate that in sub Saharan Africa about 0.7–4.3% of women exchange sex for money or goods [7]. In Uganda, 3.3% of women aged 15 and above were estimated to be FSWs in the capital city of Kampala [8]. Due to the criminalized nature of sex work in Uganda [9] these figures could be underestimates. Furthermore, criminalization drives most sex work to happen within an unhealthy and unregulated working environment. In Uganda, sex workers operate within their residence, often located in the various slum areas of trading centres, in entertainment places and along busy roadsides in some parts of the country, while others get clients through phone calls. [10].

In sub-Saharan Africa, unplanned pregnancies among female sex workers are common, ranging from 28.6% in Ethiopia [11] to 69.0% in Côte d’Ivoire [12]. Factors associated with unplanned pregnancy among FSWs include having four or more children, being unmarried, being adolescents or being older than 30 years, and use of drugs and alcohol [11, 13]. Other factors that have been associated with pregnancy planning among women in the general population include family support for becoming pregnant, residing in urban setting, and number of previous pregnancies [14].

Use of contraceptives by FSWs has been modest according to previous studies in Uganda [15]. Many times FSWs use unreliable methods such as natural methods while others use condoms inconsistently [16]. FSWs have high unmet need for sexual and reproductive health services including contraceptives due to accessibility difficulties [15]. The Ministry of Health in Uganda developed guidelines to ensure provision of integrated health services including family planning services among most at risk populations [17] but little is known about the extent to which FSWs have used contraceptives.

Pregnancy planning is a complex construct [18] and has been assessed using various ways. Most tools used in assessment assume that women already have an established choice about getting pregnant [19] yet many women are ambivalent. The London Measure of Unplanned Pregnancy (LMUP) [20], which is a psychometric measure of pregnancy planning and allows women to express a variety of positions in relation to the concept, has been used to measure pregnancy intendedness in the general population globally but has not been used among FSWs, a population at high risk of unintended pregnancies. Further, there is limited information on the extent of pregnancy planning among FSWs especially in Uganda. This study investigated contraceptive use and prevalence and predictors of pregnancy planning among FSWs in Uganda using the validated Ugandan LMUP.

## Methods

### Study design and site

A cross-sectional study design was used. Participants were recruited from four hospitals where clinics for the ‘most at risk populations initiative’ (MARPI), including FSWs, have been established, in the four regions of Uganda including Central, Northern, Western and Eastern. The MARPI clinic in central region was established in 2008 while the other three clinics were established in 2015. The MARPI clinics offer free reproductive health services including Human Immunodeficiency Virus (HIV) testing, prevention, treatment, care, support and management of other sexually transmitted infections; cancer screening, and family planning services [21]. The clinics do not provide maternal health care services.

### Sample size

A sample size of 379 women was calculated using the formula for single population cross-sectional studies [22], and assuming a prevalence of unplanned pregnancy of 44%, a 5% level of significance, and an error of 0.05. The estimated prevalence of unplanned pregnancy estimate of 44% was obtained from a study conducted among FSWs in 2012 in Gulu district in Uganda [15]. The calculated sample size was adjusted with a design effect of 2.0 to compensate for inter-cluster variation to obtain a sample size of 758. This was further adjusted for an anticipated non-response rate of 5% on any study variables [23], to obtain a final sample size of 800 participants. However data was collected among 819 FSWs due to concurrent enrolment across sites.

### Sampling procedure

The calculated sample size was allocated to the four MARPI clinics in proportion to their registered FSWs clientele. The FSWs recruited from central, Eastern, Western and Northern MARPI clinics were 517, 90, 112, and 100 respectively. FSWs were eligible for inclusion into the study if they were aged between 15 and 49 years and had been pregnant within the 2 years preceding the date of interview for this study. In addition, FSWs who could not consent because of illness or intoxication with alcohol and/or drugs at the time of screening were excluded.

MARPI Clinic in the central region receives about 20–30 FSWs per day whereas the other three sites receive 8–15 FSWs per day. We planned to enrol 6 FSWs from MARPI Clinic in the central region and 2 from the other three MARPI clinics per day over a period of 4 months. To enrol the required number of FSWs over the 4 months’ period, systematic sampling was used where every third FSW registering at the reception at MARPI Clinic in the central region and every second FSW in the other MARPI clinics were approached by research assistants. They introduced the study and obtained written informed consent to administer the screening tool. After screening, written informed consent was sought from eligible FSWs. The study was conducted between May and August 2017.

### Study tool development and pre-testing

We used the LMUP tool [20] which has been psychometrically validated in general populations in both low and high income countries [13, 19, 24–26]. The LMUP tool was reviewed and other variables found in literature to be associated with pregnancy planning were added to develop the questionnaire. The LMUP was translated into the local languages of Luganda, Acholi, Lugisu, and Runyankole and was then validated among FSWs. The revised questionnaire was further pre-tested among FSWs who had not sought services from any MARPI clinics in the district of Mukono, east of Kampala District. During pre-testing, we checked for the understanding of the various questions by FSWs and made the necessary revisions in the wording. The final pre-tested questionnaire was used to collect data by experienced research assistants who had been trained for 3 days before data collection.

### Measurements

The LMUP comprises six questions capturing information on a woman’s circumstances during the most recent pregnancy with respect to use of contraceptives, timing of pregnancy, pregnancy intention, wanting to have a baby, discussion with the man who fathered the last pregnancy, and preconception preparation. Each question was scored on 0–2 scale, with a total score of 0–12 [20]. Each point increase represents an increase in pregnancy planning effort.

Data were collected on

- Socio-demographic and economic characteristics
  We included age, marital status, and education. We also collected data on economic indicators including household properties such as ownership of a radio, television, bicycle, motorcycle, home ownership, cell phone, regular phone, computer, an income generating activity, an indoor bathroom, water source, electricity, car, generator, and solar power source.
- Sexual and obstetric history
  These included the number of living biological children, duration of working as a FSW and main place of recruiting clients. The main work place was defined as the venue for recruiting clients including streets, entertainment places, residence as well as use of phone. FSWs were also asked if they had ever tested for HIV and the most recent result. Where available, clinic records were used to classify the HIV status of each FSW. Clinic records were used in preference to the self-reported status by the FSW. In case records were missing, HIV status was categorised as unverified. Data were collected on partner type including emotional and non-emotional client (either regular or occasional clients). An emotional partner was defined as a man with whom a FSW felt an emotional attachment after a sexual encounter even if he did not give money or gifts all the time after sexual intercourse [27]. A non-emotional partner was defined as a paying client towards whom the FSW felt no emotional attachment, or a rapist. Rape was defined as forceful non-consensual penile-vaginal sexual activity during the last 2 years. Data were collected on contraceptive use and consistent condom use with paying clients. Consistent condom use with paying clients was defined as using condoms all the time with men who paid for vaginal sex.
- Social support and substance abuse
  The FSWs were asked if they had someone to provide social support in case they wanted to get pregnant. Social support was defined as any support (emotional, informational, affectionate, tangible and positive social interaction) provided by trusted and reliable person [28]. The supporters were categorised as friend, relative, health provider and no supporter. Data were also collected on hazardous alcohol use based on “The Alcohol Use Disorders Identification Test” (AUDIT) Score ≥ 7 [29]; and ever abuse of drugs.

### Data management and statistical analysis

Completed questionnaires were stored in lockable cabinets with access to only authorised study staff. Double data entry was done using EpiData software. Data were then exported to STATA version 14.0 for analysis.

The socio-demographic characteristics of participants are described using frequencies with corresponding percentages or as medians with corresponding inter quartile ranges (IQR), or as means with corresponding standard deviations, as appropriate. By using principal component analysis, five wealth quintiles were built from household properties as a measure of socio-economic status.

**Fig. 1:**
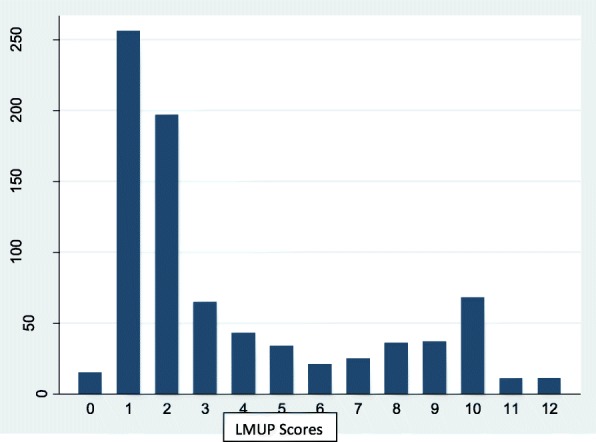
Distribution of LMUP scores among female sex workers attending MARPI Clinics

Because the LMUP scores exhibited a bi-modal distribution (see Fig. 1), scores were grouped into three categories, with scores from 0 to 3 categorized as “unplanned” pregnancy, scores from 4 to 9 categorized as “ambivalent” pregnancy planning, and scores from 10 to 12 categorized as “planned” pregnancy. This categorization is consistent with published advice on use of the LMUP tool [20, 30]. The prevalence of pregnancy planning was calculated as the percentage in each of the three categories. Statistical analyses included the chi-square test or Fisher’s exact test, to assess the statistical significance of the association between the different categories of pregnancy planning and each of the independent variables. Additionally, for purposes of comparison with previous studies that considered pregnancy planning as a binary outcome [31], FSWs with LMUP scores less than 10 (including both unplanned and ambivalent) were considered as “unplanned pregnancy category”.

Potential predictor variables investigated included those identified from the literature that have been found to be associated with pregnancy planning [13, 32]. The multinomial logistic regression model was used to estimate the variations in the probability of planning for a pregnancy across the categories. The model assumes independence among the dependent variable choices [33]. The dependent variable was “pregnancy planning status” with three categories including unplanned (0–3), ambivalent (4–9), and planned (10–12). Then, we estimated the relative risk ratios with corresponding 95% confidence intervals (95% CI) for all independent variables per category of the dependent variable with exception of unplanned pregnancy which was considers as reference category. As a first step, all variables were included in the model, and then manual stepwise backward elimination was used to remove variables not significantly associated with pregnancy planning. Variables were removed one at a time, starting with those with the largest *p*-value, until only variables significantly associated with the outcome were left in the model. A 5% level of statistical significance (α = 0.05) was used to retain variables significantly associated with pregnancy planning. Further, we investigated if any of the variables removed from the model confounded the relationship between any of the variables significantly associated with the outcome by checking if putting back such variables in the model changed the odds ratio of any of the variables retained in the model by at least 10%. If putting back any variable in the model changed the relative risk ratio by at least 10%, the variable was retained in the model regardless of its strength of association with the outcome variable. Each variable was investigated for confounding one by one.

## Results

### Number of participants

Overall a total of 925 FSWs attending MARPI clinics were screened for this study, out of which 106 (11.5%) were ineligible. Among the ineligible, 3 were beyond 49 years, 99 reported no pregnancy within past 2 years, 40 had no sex for money or gifts, and 37 were intoxicated with either alcohol or drugs and could not consent even after resting for some hours (there were multiple reasons why some women were ineligible). The remaining 819 (88.5%) eligible FSWs were enrolled, interviewed, and included in the analysis for this study.

### Characteristics of participants

The overall median age of the FSWs interviewed was 27 years (IQR 23–30), ranging from 15 to 47 years. Of all participants, 448 (54.7%) had attained primary education and 272 (33.2%) had other sources of income besides sex work. Only 116 (14%) FSWs reported were married or in union, while 480 (58.6%) reported having an emotional partner as the man who fathered the most recent pregnancy before the study. There were 243 (29.7%) HIV positive FSWs and 172 (21%) FSWs were raped in past 2 years. Hazardous alcohol users were 462 (56.5%) while 335 (40.9%) reported drug abuse. The other characteristics of the participants are summarized in Table 1.

**Table 1:** A summary of selected characteristics of the female sex workers enrolled in the study

Characteristics (*N* = 819)	Categories	Frequency (n)	Percentage (%)
Socio and economic			
Age group	15-19 yrs	42	5.1
	20-29 yrs	465	56.8
	> 30 yrs	312	38.1
Education	No education	42	5.0
	Attended Primary (1–7 years) of education)	448	54.7
	Attended Secondary or higher (at least 8 years of education)	329	40.2
Marital status	Never married	221	27.0
	Married or in union	116	14.2
	Divorce/separated /widow	482	58.8
Wealth quintile	Lowest	178	21.7
	Second	188	23.0
	Middle	135	16.5
	Fourth	196	23.9
	Highest	122	14.9
Other source of income	Yes	272	33.2
Sexual and obstetric history			
HIV status	Positive	243	29.7
	Negative	563	68.7
	Not sure/unverified by record	13	1.6
Years in sex work	< 2 years	249	30.4
	3–5 years	313	38.2
	> 6 years	257	31.4
Main workplace	Street based	270	33.0
	Entertainment place	226	27.6
	Residence/homed based	41	5.0
	Phone based	282	34.4
Partner for last pregnancy	Emotional partner	480	58.6
	Non Emotional partner	339	41.4
Number of living Children	No Child	380	46.4
	1–3 children	357	43.6
	Four and more Children	82	10.0
Ever been raped in the last two years	Yes	172	21.0
Having a supporter & substance abuse			
Reported having people to provide social support if they wanted to get pregnant	Friend	234	28.6
	Family member	180	22.0
	Health worker	172	21.0
	No support	233	28.4
Hazardous alcohol user	Yes	462	56.4
Substance use	Yes	335	40.9

### Contraceptive methods and condom use

Overall, 803 (98.0%) of the participants ever used any contraceptives and 698 (85.3%) were current users. The male condom was the most common method among users 746 (91.0%), though consistent condom use with paying client in the last month was lower at 594 (72.5%). Use of natural methods was reported by 200 (24.4%) while 475 (58.0%) used dual contraception. Though, 556 (67.9%) had heard about emergency contraceptives, only 40.6% of the 172 who were raped, accessed emergency contraception (Table 2).

**Table 2:** Knowledge and practices of contraceptive methods and condom use by FSWs attending MARPI clinics

Characteristic (*N* = 819)	Frequency (n)	Percentage (%)
Ever used any method to delay pregnancy		
Yes	803	98.0
No	16	2.0
Current users		
Yes	698	85.3
No	76	9.3
Currently pregnant	45	5.4
Current methods used^a^		
Pill	71	8.7
Injectable	308	37.6
Implant	109	13.3
Male condoms	746	91.0
Female condoms	149	18.2
Natural methods (lactation amenorrhea, rhythm and withdrawal)	200	24.4
Emergency contraception	25	3.1
Ever heard about emergency contraception		
Yes	556	67.9
No	263	32.1
Ever used dual method (condom & modern method)		
Yes currently using	475	58.0
Ever used but not currently using	140	17.1
Never	166	20.3
No answer	38	4.6
Consistent condom use with paying client in last month		
Yes	594	72.5
No	225	27.5
Condom use with last paying client		
Yes	748	92.5
No	71	7.5

^a^Multiple responses were given

### Levels of pregnancy planning

Of the 819 FSWs, 90 (11%) had planned for pregnancy (LMUP scores 10–12), 196 (23.9%) were ambivalent (LMUP score 4–9), and 533 (65.1%) did not plan the pregnancy (LMUP scores 0–3). When pregnancy planning was considered as binary outcome, 729 (89%) had unplanned pregnancies (0–9 scores). There was a fairly equal distribution of the proportion of FSWs who planned pregnancies, across wealth quintiles (except middle), and alcohol use categories. Other variables were not significant. The distribution of FSWs at different LMUP cut off points by selected independent variables is illustrated in Table 3.

**Table 3:** Planning status of pregnancy by selected characteristics of female sex workers

Characteristics (*N* = 819)	Unplanned *N* = 533 n (%)	Ambivalent *N* = 196 n (%)	Planned *N* = 90 n (%)	*P* value Chi2 (x^2^ test)
Age group				*P*= 0.024*
15-19 yrs	21 (3.9)	15 (7.6)	6 (6.7)	
20-29 yrs	291 (54.6)	115 (58.7)	59 (65.5)	
> 30 yrs	221 (44.5)	66 (33.7)	25 (27.8)	
Marital status				*P*= 0.230
Never married	145 (27.2)	50 (25.5)	26 (28.9)	
Married or in union	65 (12.2)	34 (17.4)	17 (18.9)	
Divorce/separated /widow	323 60.6)	112 (57.1)	47 (52.2)	
Wealth quintile				*P*= 0.016*
Lowest	128 (24.0)	32 (16.3)	19 (20.0)	
Second	129 (24.2)	38 (19.4)	21 (23.3)	
Middle	88 (16.5)	38 (19.4)	9 (10.0)	
Fourth	125 (23.5)	49 (25.0)	22 (24.5)	
Highest	63 (11.8)	39 (19.9)	20 (22.2)	
HIV status				*P*= 0.618
Positive	160 (30.0)	62 (31.6)	21 (23.3)	
Negative	364 (68.3)	132 (67.4)	67 (74.5)	
Not sure/unverified by records	9 (1.7)	2 (1.0)	2 (2.2)	
Years in sex work				*P*= 0.004*
< 2 years	141 (26.4)	73 (37.2)	35 (38.9)	
3–5 years	204 (38.3)	74 (37.8)	35 (38.9)	
> 6 years	188 (35.3)	49 (25.0)	20 (22.2)	
Main workplace				*P* = 0.218
Street based	174 (32.7)	65 (33.2)	31 (34.4)	
Entertainment place	151 (28.3)	45 (23.0)	30 (33.3)	
Residence/homed based	25 (4.7)	9 (4.6)	7 (7.8)	
Phone based	183 (34.3)	77 (39.3)	22 (24.4)	
Partner for last pregnancy				*P*= 0.000*
Emotional partner	242 (45.4)	160 (81.6)	78 (86.7)	
Non emotional (Paying client/others)	291 (54.6)	36 (18.4)	12 (13.3)	
Number of living Children				*P* = 0.206
No child	249 (46.7)	91 (46.4)	40 (44.4)	
1–3 children	222 (41.7)	90 (45.9)	45 (50.0)	
≥ 4 children	62 (11.6)	15 (7.65)	15 (5.6)	
Ever been raped in last two years				*P*= 0.000*
-No	398 (74.7)	171 (87.2)	78 (86.7)	
-Yes	135 (25.3)	25 (12.8)	12 (13.3)	
Reported having person/s to provide social support				*P*= 0.000*
Friend	134 (25.1)	67 (34.2)	33 (36.7)	
Family member	99 (18.6)	53 (27.0)	28 (31.1)	
Health worker	121 (22.7)	37 (18.9)	14 (15.5)	
No support	179 (33.6)	39 (19.9)	15 (16.7)	
Alcohol use				*P*= 0.025*
Non-Hazardous drinking	214 (40.2)	97 (49.5)	46 (51.1)	
Hazardous drinking	319 (59.8)	99 (50.5)	44 (48.9)	
Substance use				*P*= 0.002*
No	291 (54.6)	132 (67.3)	61 (67.8)	
Yes	242 (45.4)	64 (32.7)	29 (32.2)	

*Significant statistical difference between groups, Pearson`s Chi-square or Fisher`s test (*P*< 0.05)

### Predictors of pregnancy planning

FSWs with a non-emotional partner as a man who impregnated her, were less likely to plan for a pregnancy by 0.15 times in the planned pregnancy category and 0.22 times in the ambivalent category, keeping other variables in the model constant. Similarly lack of social support reduced the level of pregnancy planning by 0.44 times among those in planned category and 0.54 times in the ambivalent category when other variables were held constant. Rape in the last 2 years and abusing substances significantly influenced planning of pregnancy among participants in the ambivalent group and not in the planned category. Holding other variables constant, the risk of being in ambivalent group versus unplanned group was 0.51 times less for participants who had ever been raped in the last 2 years relative to those who reported being never raped. Lastly the FSWs who ever abused substances were 0.65 times less likely to plan for pregnancy in the ambivalent category compared to those who did not abuse substances keeping all variables in the model constant. All variables that were significant in the final model are shown in Table 4.

**Table 4:** Multinomial logistic regression analysis showing factors associated with pregnancy planning

Variables/categories	Planned Pregnancies		Ambivalent Pregnancies	
	RRR	95% CI	RRR	95% CI
Partner for last pregnancy				
Emotional partner (rc)	1.00	–	1.00	–
Non emotional (Paying client/others)	*0.16	(0.08–0.30)	*0.22	(0.15–0.33)
Reported having person/s to provide social support				
Friend (rc)	1.00	–	1.00	–
Family member	1.08	(0.60–1.95)	1.01	(0.62–1.61)
Health worker	0.54	(0.27–1.09)	0.68	(0.42–1.13)
No support	*0.44	(0.22–0.87)	*0.54	(0.33–0.88)
Ever been raped in last two years				
No (rc)	1.00	–	1.00	–
Yes	0.55	(0.28–1.07)	*0.51	(0.31–0.84)
Substance use				
No (rc)	1.00	–	1.00	–
Yes	0.63	(0.38–1.04)	*0.61	(0.44–0.93)

Note: *rc* Reference category, *LR chi square* 150.68, *Pseudo R*^*2*^ 10.6%, *base model* Unplanned pregnancy; *means variable is significant at *p* < 0.05

## Discussion

Our study shows a low prevalence (11.0%) of planned pregnancies among FSWs in Uganda. This is comparatively lower than the 44% which was reported in another study in northern Uganda [15] and the 59% among women in the general population [23]. The observed low prevalence could be because of more nuanced measurement using the LMUP tool. We were able to categorise pregnancy planning into three groups, rather than two. This included ambivalent status which allows women to express their indecisiveness about pregnancy planning. Indeed, a substantial percentage of the participants (23.9%) in this study were ambivalent about pregnancy planning. Previous studies have categorised planning status into binary outcome of planned and unplanned pregnancy which assumes that women have clarity on whether to get pregnant or not before conception.

The overall low proportions of FSWs with planned pregnancies could be attributed to poor utilization of reliable family planning methods. In this study though about 85% of the FSWs reported to be currently using a method to delay pregnancy, many used methods with high failure rates. We observed high proportions (91.0%) reporting condom use but consistent use was 72%. Inconsistent condom use is less effective as a pregnancy prevention tool and may have predisposed FSWs to unplanned pregnancies [34]. Others reported using natural methods, and dual use of contraceptives was at only 58%. A previous study in Uganda [15] and other countries in Africa [35] indicated low dual contraceptive use, even among FSWs with no intention of getting pregnant. Dual contraception with a condom and other effective contraceptive would be ideal for this population which is at high risk of pregnancy and other sexually transmitted infections including HIV [15] .

We observed that FSWs who had been raped within the previous 2 years were less likely to have planned their most recent pregnancy. We also observed that only 40% of the women who were raped accessed emergency contraception (EC). Failure to use EC after unprotected sex leads to unplanned pregnancies among FSWs not using modern contraceptives. Similarly, a cross-sectional survey conducted in Gambia in 2015, found high levels of unplanned pregnancies among FSWs who had experienced sexual violence [36].

In this study, no association was observed with alcohol and pregnancy planning in the adjusted analysis. However, an association between drug use and reduced pregnancy planning was found. This could be linked to failure to negotiate for condom use with clients after getting intoxicated with drugs as was noted in other low income countries [37]. Similarly, the Gambia study found that alcohol had no effect on the ability to negotiate for condom use among FSWs with patrons. There is no clear explanation as to why alcohol use had no effect yet drug use reduced the odds of pregnancy planning in either this or our study. However, in this study we excluded FSWs who were intoxicated with alcohol and other substances at screening, which could potentially contribute to this finding. A qualitative study among a cohort of FSWs in Uganda indicated that clients usually take advantage of drunken FSWs to have unprotected sex [38]. Since there are many participants who reported to be using drugs or taking alcohol in this study there is need to intensify intervention for harm reduction.

Low levels of pregnancy planning were observed among FSWs who had non-emotional partner as the man who fathered the last pregnancy. Most of the non-emotional partners were paying clients. This could be attributed to failure to use condoms consistently and correctly as a result of rushing negotiations with non-emotional partners among FSWs given that sex work is illegal and attracts penalties [9, 39]. A study conducted among FSWs in Gulu in northern Uganda found that rushing negotiation with clients due to police presence was negatively associated with dual contraceptive use [15]. Inadequate time to negotiate condom use and low utilization of modern contraception leads to unplanned pregnancies.

Again we observed low levels of pregnancy planning among participants who had no person to provide social support. A study among a cohort of family planning clients in Kenya showed that women receiving health services from providers offering high levels of quality of care are more likely to plan for pregnancies [40]. However, in this study we did not assess the quality of care at the MARPI clinics. Social support shapes pregnancy experiences among women especially in low income countries with high rates of unintended pregnancy [41]. Women who receive appropriate social support are likely to have reduced rates of induced abortions [42]. However, further studies are needed to better understand the actual support provided by different individuals in order to devise interventions to reduce induced abortions among FSWs.

No significant association was observed between planned pregnancies and HIV status. Though HIV status was self-reported and cross checked with records, we observed a higher HIV prevalence of 29.7% among the FSWs in this study compared to 7.6% among women in the general population [43]. A study conducted in study in Abidjan, Côte d’Ivoire in 2015 found no significant difference in the proportion of unplanned pregnancies among HIV infected and uninfected FSWs [12]. Due to regular contact with health facilities as women seek HIV treatment and care, one would expect HIV infected FSWs to plan better for pregnancies. However this may be limited by the generally low integration of safer conception support in HIV care [44]. More interventions are required to control the acquisition and spread HIV among FSWs in Uganda as well as focusing on pregnancy planning.

### Strengths and limitations

This is the first study to our knowledge that has assessed pregnancy planning among FSWs using the LMUP tool. The use of LMUP instead of a one-question method is a strength, and this may have helped to reduce misclassification of unplanned pregnancies [13]. In addition, this paper draws strength from the systematic selection of participants at the MARPI clinics in the four regions of the country (Central, Eastern, Northern and Western). This implies that FSWs attending MARPI clinics had equal chances of being selected. We included pregnant, post-partum and FSWs who had had abortions, catering for all possible pregnancy outcomes. Though some literature has shown differing pregnancy intendedness after delivery [45] a longitudinal study conducted in India using the LMUP found no increase in reported pregnancy intention after 2 year of follow up [24]. This implies results can be applied to both pregnant and postpartum FSWs.

There are some limitations with our study. First our study is informed by reported behaviours. The FSWs may have inaccurately reported their sexual behaviours due to recall bias as FSWs recruited in the study could have been pregnant at any time over a two-year interval. Since most women in African settings are expected to have children, social desirability among FSWs could have contributed to over reporting of pregnancy planning. We believe our well-trained research assistants, supervised by the principle investigator who has worked with FSWs over 10 years, established strong rapport and trust among the FSWs to provide accurate information. Lastly we cannot interpret the temporal relationship between independent variables and pregnancy planning among FSWs because of the cross sectional study design used. Since the sample included in the study was clinic based, the findings may not be generalizable to all sex workers in Uganda.

## Conclusion and recommendation

We found low prevalence of planned pregnancies among FSWs compared to women in the general population in Uganda amidst low and inconsistent contraceptive use. Our results emphasize the need to design robust approaches to implement comprehensive reproductive health services among FSWs. In particular, there is urgent need to promote consistent use of reliable contraceptives among FSWs to prevent unplanned pregnancies with non-emotional partners and after rape. While our study has identified the prevalence and predictors of pregnancy planning, there is need to conduct further research to assess the impact of pregnancy intention on pregnancy outcome and utilization of maternal services among FSWs.

## Abbreviations

AUDIT: The Alcohol Use Disorders Identification Test
EC: Emergency contraception
FSWs: Female Sex Workers
HIV: Human Immunodeficiency Virus
IQR: Inter-Quintile Range
LMUP: London Measure of Unplanned Pregnancy
MARPI: Most at Risk Population Initiative
